# CD36 is a co-receptor for hepatitis C virus E1 protein attachment

**DOI:** 10.1038/srep21808

**Published:** 2016-02-22

**Authors:** Jun-Jun Cheng, Jian-Rui Li, Meng-Hao Huang, Lin-Lin Ma, Zhou-Yi Wu, Chen-Chen Jiang, Wen-Jing Li, Yu-Huan Li, Yan-Xing Han, Hu Li, Jin-Hua Chen, Yan-Xiang Wang, Dan-Qing Song, Zong-Gen Peng, Jian-Dong Jiang

**Affiliations:** 1 Institute of Medicinal Biotechnology, Chinese Academy of Medical Sciences & Peking Union Medical College, Beijing, 100050, China; 2 State Key Laboratory of Bioactive Substance and Function of Natural Medicines, Institute of Materia Medica, Chinese Academy of Medical Sciences & Peking Union Medical College, Beijing, 100050, China

## Abstract

The cluster of differentiation 36 (CD36) is a membrane protein related to lipid metabolism. We show that HCV infection *in vitro* increased CD36 expression in either surface or soluble form. HCV attachment was facilitated through a direct interaction between CD36 and HCV E1 protein, causing enhanced entry and replication. The HCV co-receptor effect of CD36 was independent of that of SR-BI. CD36 monoclonal antibodies neutralized the effect of CD36 and reduced HCV replication. CD36 inhibitor sulfo-N-succinimidyl oleate (SSO), which directly bound CD36 but not SR-BI, significantly interrupted HCV entry, and therefore inhibited HCV replication. SSO’s antiviral effect was seen only in HCV but not in other viruses. SSO in combination with known anti-HCV drugs showed additional inhibition against HCV. SSO was considerably safe in mice. Conclusively, CD36 interacts with HCV E1 and might be a co-receptor specific for HCV entry; thus, CD36 could be a potential drug target against HCV.

Hepatitis C virus (HCV) is a hepatotropic positive-sense single-stranded RNA virus which belongs to *Flaviviridae* family and is one of the major causes for chronic hepatitis and liver disease worldwide^1^. Since the identification of HCV in 1989, the life cycle and replication mechanism of the virus have been illustrated, and a number of cell surface factors that help HCV entry have been identified^2^. Accumulated data suggest that HCV entry is a complex and multistep process. Non-specific host receptors glycosaminoglycans (GAGs)^3^ and the low-density lipoprotein receptor (LDL-R) may facilitate initial attachment of HCV particles on the cell surface^4^. HCV particle appears to interact with a series of cell membrane proteins, including tetraspanin CD81^5^, scavenger receptor class B member I (SR-BI)^6^, tight-junction proteins claudin-1^7^ and occludin^8^, followed by clathrin-mediated endocytosis and fusion between the virion envelope and endosomal membrane^9^,^10^. Building on the knowledge of these co-factors, Dorner M *et al.* established a humanized mouse model for HCV infection^11^. However, Hikosaka K *et al.* showed that expression of human factors CD81, claudin-1, scavenger receptor and occludin in mouse hepatocytes could not confer susceptibility to HCV entry^12^. Another group showed that Tupaia CD81, SR-BI, claudin-1 and occludin supported HCV infection^13^. Recently, Dorner M *et al.* completed their demonstration on the entire HCV life cycle in genetically humanized mice^14^. These data suggest the existence of unknown cellular factors that help HCV to enter host cells. New host factors co-facilitating HCV particles entry were discovered in the past few years, such as tyrosine kinases epidermal growth factor receptor^15^, ephrin receptor A2^15^, the cholesterol uptake receptor Niemann–Pick C1 like 1^16^, transferrin receptor 1^17^ and SR-BI partner PDZK1^18^. The findings provide new information to clarify the detailed mechanism for HCV entry.

Our group has a long history of doing research on compounds that regulate lipid metabolism, in which we found recently that antagonists for cluster of differentiation 36 (CD36) significantly reduced HCV replication in human hepatocytes. The finding caused our interest in the function of this molecule in HCV infection. CD36 is a transmembrane protein and its function is mainly associated with lipid metabolism^19^, but its role in HCV infection is unknown. By using CD36 inhibitors as chemical probes we found that CD36 appears to be another co-factor assisting HCV for attachment on and entry into host cells; blocking the effect of CD36 significantly inhibited HCV replication.

## Results

### CD36 expression was up-regulated in HCV-infected hepatocytes

**Figure 1:**
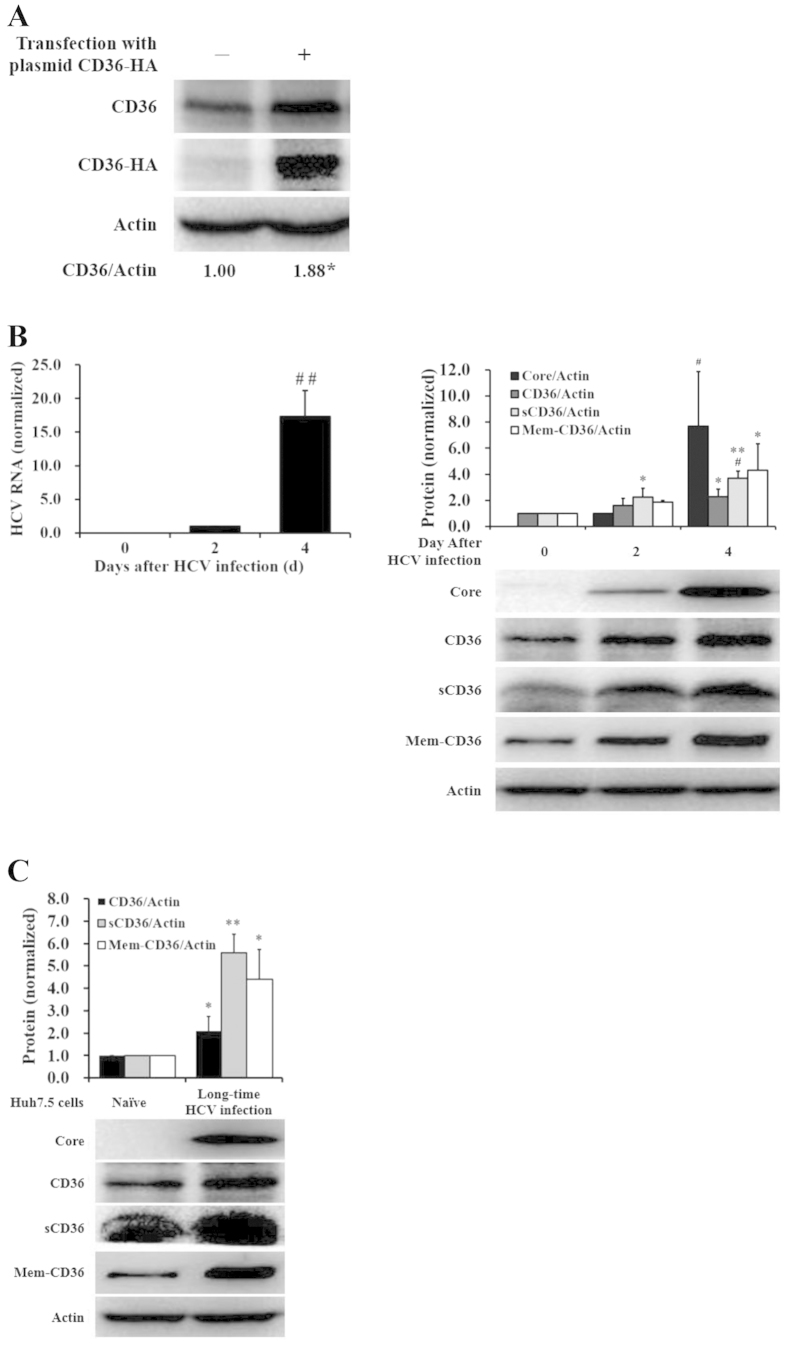
HCV infection increased CD36 expression *in vitro.* (**A**) CD36 expression on naïve Huh7.5 cells was confirmed with the band at the site of exogenous CD36-HA detected with anti-HA antibody. The ratio of CD36/Actin is the average taken from 4 independent experiments (*P < 0.05 *vs* plasmid control (−)). (**B**) HCV infection increased CD36 expression on Huh7.5 cells and elevated sCD36 in culture supernatants (*n* = 3; *P < 0.05 and **P < 0.01 *vs* day 0; ^#^*P* < 0.05 *vs* day 2). (**C**) CD36 expression and sCD36 secretion were increased on Huh7.5 cells infected with HCV for over 60 days (*n* = 3; *P < 0.05 and **P < 0.01 *vs* naïve control). Huh7.5 cells were infected with HCV (45IU/cell), proteins and intracellular HCV RNA were respectively detected with WB and qRT-PCR at indicated days after infection in (**B**,**C**). The protein bands presented in the figure showed the results of a representative experiment. Data presented are mean ± standard deviation. *Student’*s *t*-test was used.

CD36 expresses on several types of mammalian cells, such as platelets, erythrocytes, monocytes, differentiated adipocytes, skeletal muscle, mammary epithelial cells, skin microdermal endothelial cells, and hepatocytes as well^20^,^21^. To learn CD36 expression on human liver Huh7.5 cells, which are sensitive to HCV infection^22^, naïve Huh7.5 cells were transfected with CD36-expression vector fusing HA tag at the C-terminus, followed by western blot detection. Figure 1A showed that CD36 indeed expressed on the Huh7.5 cells with the protein size almost consistent with that of exogenous CD36-HA, and the total CD36 expression increased after transfection with exogenous CD36-HA plasmid (Fig. 1A, *P* < 0.05). Huh7.5 cells infected with HCV showed a time-dependent increase of CD36 expressed on cell surface (Mem-CD36) (Fig. 1B, right, *P* < 0.05 for Mem-CD36), which was consistent with the total level of CD36 expressed on Huh7.5 cells (Fig. 1B, right, *P* < 0.05 for CD36), and the level of soluble CD36 (sCD36) in culture supernatants was elevated accordingly after HCV infection (Fig. 1B, right, *P* < 0.05 on day 2, *P* < 0.01 on day 4); the elevations were positively correlated with HCV replication (Fig. 1B, *P* < 0.05 for HCV Core (right) and RNA (left)). Furthermore, CD36 expression on hepatocytes or secretion of sCD36 in supernatants was elevated in the Huh7.5 cells infected with HCV for over 60 days (Fig. 1C, *P* < 0.05 for CD36, *P* < 0.01 for sCD36, *P* < 0.05 for Mem-CD36). The results agreed with that observed in clinic, showing elevated CD36 expression on liver cell and increased sCD36 concentration in sera in HCV-infected individuals^23^,^24^,^25^. Thus, HCV infection appeared to be a direct cause for CD36 up-regulation.

### HCV replication was enhanced by CD36

**Figure 2:**
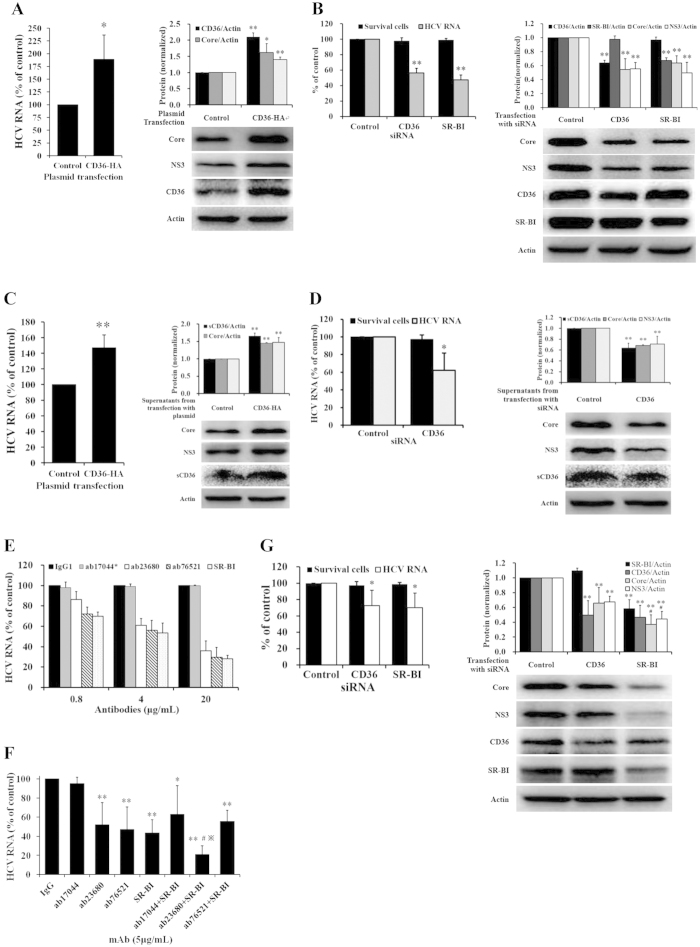
HCV replication was increased by CD36. (**A**) High expression of CD36 increased HCV infection and replication (*n* = 3). (**B**) Silence of CD36 decreased HCV infection and replication (*n* = 3). Huh7.5 cells were transfected with 0.2 μg CD36-HA plasmid (**A**) or with 150 pmol siRNA for CD36 (**B**) for 48 hrs, and then were washed and infected with HCV (150 IU/cell) for 2 hrs, followed by washing and continuously incubating. Intracellular HCV RNA and proteins were detected in 96 hrs, and the cytotoxicity of siRNAs was measured in 96-wells plate with a MTT assay in **B**. (**C**) High sCD36 in supernatants increased HCV infection (*n* = 3 for RNA, *n* = 4 for protein). (**D**) Low sCD36 in supernatants decreased HCV infection (*n* = 3 for RNA and cytotoxicity, *n* = 4 for protein). Huh7.5 cells were incubated with culture supernatants of Huh7.5 cells transfected with 0.2 μg CD36-HA plasmid (**C**) or with 150 pmol siRNA for CD36 (**D**) for 48 hrs, and infected with HCV (150 IU/cell) for 2 hrs, followed by washing and continuously culturing. Intracellular HCV RNA and proteins were detected in 72 hrs, and cytotoxicity was measured with a MTT assay accordingly in **D**. The pcDAN3.1(+) vector was used as plasmid control in **A** and **C**. The SR-BI siRNA (sc-44753) was used as positive control and siRNA-A as negative control in (**B**). *P < 0.05 and **P < 0.01, *vs* control; ^#^*P* < 0.05, *vs* CD36 siRNA. (**E**) CD36 mAbs neutralized HCV infection in a dose-dependent manner (concentrations of ab17044 were 0.2, 1, and 5 μg/mL) (*n* = 3). (**F**) CD36 mAbs synergistically inhibited HCV infection with SR-BI antibody (*n* = 4). Huh7.5 cells were incubated with 5 μg/mL mAb for 1 hr and then infected with HCV (150 IU/cell) for 2 hrs, followed by washing and culturing. Intracellular HCV RNA was detected in 72 hrs (**E**,**F**). *P < 0.05 and **P < 0.01, *vs* IgG group; ^#^,*P* < 0.05, *vs* monotherapy with ab23680 or SR-BI antibody. The mAbs code was from Abcam, Co. Ltd. (**G**) Cross-silencing test of CD36 and SR-BI (sc-44752), and cytotoxicity was measured with a MTT assay (*n* = 3 for RNA and cytotoxicity, *n* = 6 for protein). The protein bands presented in the figure showed the results of a representative experiment. Presented are mean ± standard deviation. *Student*’s *t*-test was used in (**A–G**).

To learn the role of CD36 in HCV infection, we first examined whether CD36 could affect HCV replication *in vitro*. Huh7.5 cells were transfected with CD36-HA plasmid. 48 hrs after transfection, the cells were washed and then infected with HCV for 2 hrs, following by washing with PBS and culturing with fresh medium. Intracellular HCV RNA and proteins was detected in 96 hrs. As shown in Fig. 2A, with the increase of CD36 (Fig. 2A, right, *P* < 0.01), intracellular HCV RNA (Fig. 2A, left, *P* < 0.05) and proteins (Fig. 2A, right, *P* < 0.05 for Core, and *P* < 0.01 for NS3) were significantly higher in the Huh7.5 cells transfected with plasmid CD36-HA as compared with in those transfected with plasmid control. Transfection of CD36 without HA-tag exhibited similar results (not shown), indicating that fusing HA tag at the CD36 C-terminus had no effect on its function. The results suggest an enhancing effect of CD36 in HCV replication. To validate the results, specific siRNA for CD36 was used to silence the endogenous CD36 gene expression in Huh7.5 cells. After 48 hrs transfection of siRNA for CD36, the cells were washed and then infected with HCV, followed by measurement for HCV replication. As shown in Fig. 2B, the intracellular HCV RNA load largely reduced in 96 hrs (Fig. 2B, left, *P* < 0.01) and HCV proteins (HCV Core and NS3) declined as well (Fig. 2B, right, *P* < 0.01 for Core and NS3), the positive control siRNA for SR-BI inhibited HCV replication, which is consistent with previous report^26^.

Next, we focused on the question of if sCD36 affects HCV replication. The culture supernatants of Huh7.5 cells treated 48 hrs with transfection of CD36-HA plasmid or CD36 siRNA were collected. Naïve Huh7.5 cells were cultured with the above culture supernatants and infected with HCV for 2 hrs; then, supernatants were replaced with fresh media. Intracellular HCV RNA and proteins were detected in 72 hrs. Results showed that the supernatants of Huh7.5 cells transfected with CD36-HA high-expression plasmid enhanced HCV replication (Fig. 2C, left, *P* < 0.01 for HCV RNA; right, *P* < 0.01 for HCV proteins), while the supernatants of those transfected with siRNA for CD36 reduced HCV replication (Fig. 2D, left, *P* < 0.05 for RNA; right, *P* < 0.01 for proteins). HCV replication appeared to be positively correlated with the concentration of sCD36 in supernatants (*P* < 0.01), suggesting a role of sCD36 in supporting HCV replication. It appears that HCV infection causes an increased expression and secretion of CD36, which feedbacks an enhancing effect for HCV replication.

Then, specific monoclonal antibodies (mAbs) against CD36 were used to further validate the role of CD36. The three CD36 mAbs were active against 3 CD36 epitope regions that are null of SR-BI sequence. The tests were done for the attachment and entry steps of HCV life cycle. Huh7.5 cells were treated with mAb for 1 hr and then infected with HCV; after 2 hrs infection, the culture supernatants were replaced with fresh media. Quantification of intracellular HCV RNA was performed in 72 hrs. As shown in Fig. 2E, pre-treatment of the cells with CD36 mAb (ab23680 or ab76521) significantly reduced HCV RNA in HCV-infected Huh7.5 cells in a dose-dependent manner. As a positive control, SR-BI antibody significantly reduced HCV RNA in a dose-dependent manner (Fig. 2E). However, CD36 mAb (ab17044) did not affect HCV replication even at concentration of 5 μg/mL (Fig. 2E), suggesting that the antigenic domain of this mAb on CD36 plays no role in HCV entry. These results confirm that CD36 plays a significant role in helping HCV replication at the stage of viral attachment and entry.

Considering that the SR-BI is one of the known HCV receptors^6^ and the homology in protein sequences between SR-BI and CD36 is about 30%^27^, we then further examined the joint effect of the mAbs. As shown in Fig. 2F, combination of CD36 mAb (ab23680) with SR-BI specific antibody demonstrated an additional inhibition on HCV replication at the entry stage (*P* < 0.01, *vs* IgG; *P* < 0.05, *vs* SR-BI or ab23680 alone), suggesting that the domain of CD36 molecule might help HCV entry in a way different from that of SR-BI. However, combination of CD36 mAb (ab76521) with the SR-BI antibody showed no benefit at all in blocking HCV entry, and binding competition might be part of the explanation. In addition, cross-silencing test of the two genes was done to examine the role of CD36. Transfection of specific siRNA for CD36 did not affect the expression of SR-BI (Fig. 2G, right, *P* > 0.05), whereas silencing SR-BI RNA expression with SR-BI siRNA (sc-44752) inhibited CD36 expression (Fig. 2G, right, *P* < 0.01 for CD36); and accordingly, the HCV Core or NS3 in SR-BI-silenced cells was lower than that in the CD36-silenced cells (*P* < 0.05 for Core and NS3) with no cytotoxicity was observed in this experiment (Fig. 2G, left). It appears that CD36 is a new co-factor for HCV entry, working through coordination with SR-BI.

### CD36 facilitated HCV attachment via direct interaction with HCV glycoprotein E1

**Figure 3:**
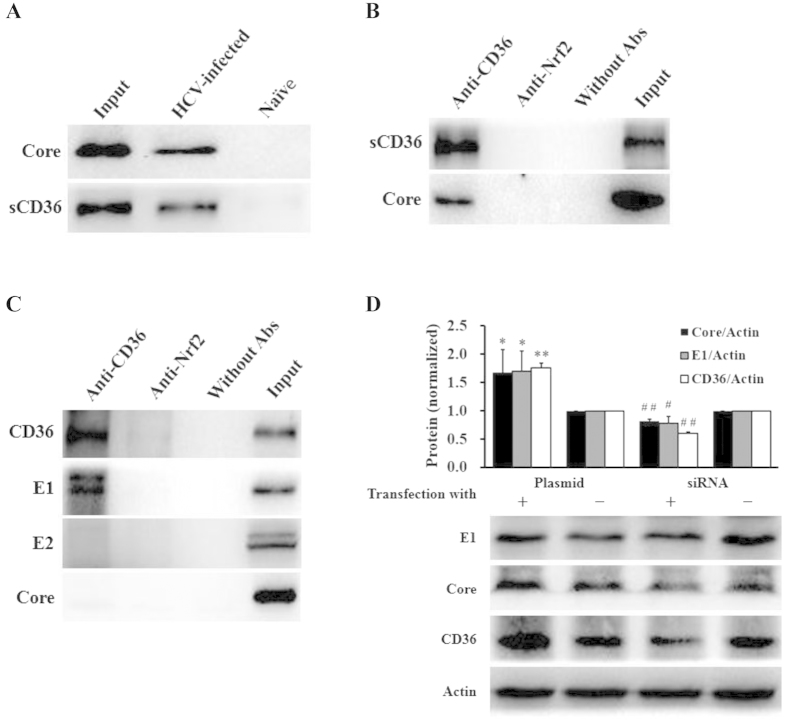
CD36 directly interacted with HCV E1 protein. (**A**) HCV particles bound sCD36 in culture supernatants. The culture supernatants of HCV-infected or naïve Huh7.5 cells were ultracentrifuged, and HCV viral components (Core) and sCD36 were simultaneously detected in the precipitates using western blot. (**B**) sCD36 bound onto HCV particles in culture supernatants (Co-IP assay). Co-IP with anti-CD36 mAb from the supernatants of HCV-infected Huh7.5 cells brought down HCV particles. (**C**) CD36 directly bound HCV E1 protein. Co-IP was performed with the lysates of HCV-infected Huh7.5 cells. Anti-Nrf2 antibody was used as an irrelevant negative control, and Without Abs as blank control in (**B**,**C**). (**D**) CD36 on cell membrane helped host to capture HCV particles (*n* = 3). After 48 hrs transfection with plasmid or siRNA for CD36, 293T/17 cells (resistant to HCV infection) were washed and incubated with HCV (300 IU/cell) for 3 hrs, followed by washing and then treating with lysis buffer. CD36 and HCV proteins in the lysates were analyzed with western blot. *P < 0.05, *vs* plasmid control (−); ^#^*P* < 0.05 and ^##^*P* < 0.01, *vs* siRNA control (−). The protein bands presented showed the results of a representative experiment. Presented are mean ± standard deviation, and *Student*’s *t*-test was used.

Next, we examined whether CD36 directly interacts with HCV particle. The culture supernatants of HCV infected Huh7.5 cells were ultracentrifuged for viral particle collection, followed by protein analysis of the pellets. As shown in Fig. 3A, HCV viral Core and sCD36 were simultaneously detected with western blot in the precipitates. In addition, we used anti-CD36 mAb to pull down HCV particles in the infectious culture supernatants with Co-IP technology; and indeed, sCD36 and HCV particles (Core) were concurrently detected in the immunoprecipitates (Fig. 3B). To learn what is the HCV proteins that directly interact with CD36, we used anti-CD36 mAb to pull down intracellular HCV components. Western blot results showed that HCV E1, but not E2 or Core, was pulled down by anti-CD36 mAb (Fig. 3C). The results were consistent with a previous report showing that HCV E2 did not bind CD36^6^.

To study whether or not the membrane CD36 could gather HCV particles to facilitate HCV entry, the 293T/17 cells, which are derived from kidney epithelial cells and resistant to HCV infection, were employed. The 293T/17 cells were transfected with CD36 expression vector or siRNA. After 48 hrs incubation, culture supernatants were replaced with fresh media and HCV viral stock was added for a 3 hrs incubation at 37 °C, followed by western blot analysis for HCV proteins in the whole cell lysates. Results (Fig. 3D) showed that HCV particles adhering onto the cell surface were increased in the cells transfected with CD36 expression plasmid (*P* < 0.05), while cells transfected with CD36 siRNA showed a decreased surface adherence of HCV particles (*P* < 0.01 for Core, *P* < 0.05 for E1), and the change was consistent with that of level of CD36. The results suggest that CD36 might directly bind glycoprotein E1 and thus increase the level of HCV particle on cell surface. It might facilitate HCV entry in the early stage of replication.

### CD36 inhibitors blocked HCV replication *in vitro*

**Figure 4:**
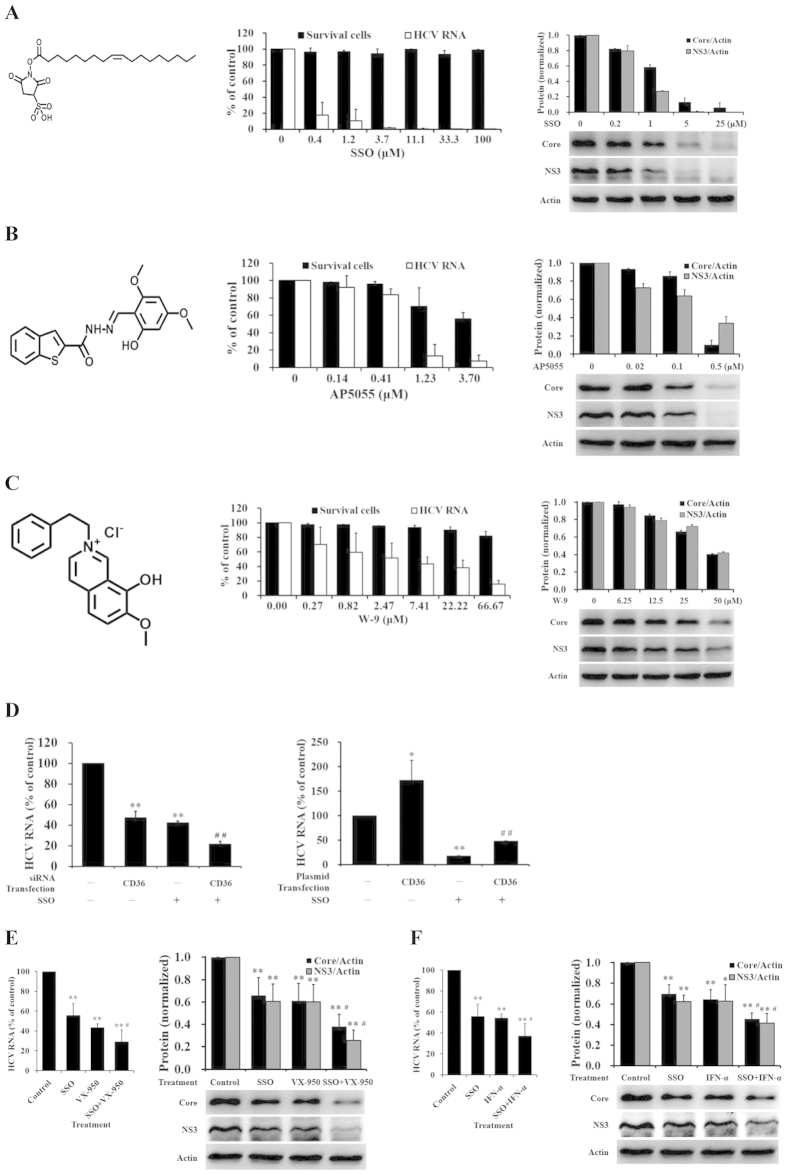
CD36 inhibitors blocked HCV replication *in vitro*. (**A**–**C**) CD36 inhibitors prevented HCV from replicating (*n* = 3). Huh7.5 cells were infected with HCV (45 IU/cell) and simultaneously treated with compound SSO (**A**), AP5055 (**B**), W-9 (**C**) or solvent control. After 72 hrs incubation, intracellular RNA and proteins were detected, and cytotoxicity was measured with MTT assay (Middle). (**D**) The anti-HCV activity of SSO was affected by the level of CD36 (*n* = 3). Huh7.5 cells were transfected with 150 pmol siRNA for CD36 (Left) or with 0.2 μg CD36 plasmid (Right). After 48 hrs, the cells were washed and infected with HCV (150 IU/cell) and simultaneously treated with 5 μM SSO for 2 hrs, followed by washing and continuously incubating with fresh media. Intracellular HCV RNA was detected in 96 hrs. *P < 0.05 and **P < 0.01, *vs* siRNA (or plasmid) control plus solvent control group; ^##^*P* < 0.01, *vs* siRNA (or plasmid) control plus SSO group. (**E**) SSO in combination with VX-950 inhibited HCV replication in an additive manner (*n* = 3 for RNA, *n* = 4 for protein). (**F**) Increased anti-HCV effect of SSO in combination with IFN-α (*n* = 3 for RNA, *n* = 4 for protein). Huh7.5 cells were infected with HCV (45 IU/cell) and incubated with SSO (1.0 μM) or VX-950 (0.1 μM), or the combination of SSO and VX-950 (**E**), or incubated with IFN-α (1.0 U/mL), or the combination of SSO and IFN-α (**F**). Intracellular RNA and proteins were detected in 72 hrs. *P < 0.05 and **P < 0.01, *vs* solvent control; ^#^*P* < 0.05, *vs* VX-950 or IFN-α alone. The protein bands showed the results of a representative experiment. Presented are mean ± standard deviation. *Student*’s *t*-test was used in (**D–F**).

There were several groups of compounds showing selective inhibitory effect on CD36^28^,^29^,^30^. If CD36 is really a co-receptor for HCV attachment and entry, those inhibitors should be potential drug candidates against HCV. We therefore selected three types of compounds independently discovered in different laboratories, sulfo-N-succinimidyl oleate (SSO)^28^ (Fig. 4A, left), AP5055^29^ (Fig. 4B, left) and W-9^30^ (Fig. 4C, left), to examine their anti-HCV activities in Huh7.5 cells. As expected, all of the three compounds displayed strong or moderate inhibition against HCV replication (Fig. 4A–C). Among these CD36 inhibitors, SSO displayed the most promising activity against HCV at RNA level, with the half maximal effective concentration (EC_50_) of 0.31 ± 0.09 μM and the selective index (SI) above 326 (Fig. 4A, middle); AP5055 was active against HCV with EC_50_ of 0.39 ± 0.16 μM but SI of 25 (Fig. 4B, middle); and the anti-HCV activity of W-9 was relative weak with EC_50_ of 6.61 ± 3.18 μM and SI of 20 (Fig. 4C, middle). Western blot examination confirmed the anti-HCV results at HCV proteins level (Fig. 4A, right; 4B, right; and 4C right). The different activities against HCV replication among these compounds might be due to their binding on different domains or site of CD36^28^,^29^,^30^. These results indicate that CD36 might be a drug target against HCV.

To confirm whether SSO inhibited HCV replication via CD36, Huh7.5 cells were transfected with siRNA for CD36 or with CD36 plasmid. After 48 hrs, the cells were infected with HCV and simultaneously treated with SSO for 2 hrs, followed by washing and continuously incubating with fresh media. Intracellular HCV RNA was detected in 96 hrs. Results showed that anti-HCV activity of SSO was increased by transfection with siRNA for CD36 (Fig. 4D, left, *P* < 0.01), while decreased by transfection with CD36 plasmid (Fig. 4D, right, *P* < 0.01), suggesting that the level of CD36 will impact the anti-HCV activity of SSO.

As SSO appears to block HCV replication through inhibiting CD36-related viral entry, an anti-HCV mechanism different from that of direct-acting antiviral agents (DAAs) and interferon-alpha (IFN-α), we combined SSO with DAAs or IFN-α to treat HCV infection. Indeed, addition of SSO enhanced anti-HCV efficacy of telaprevir (VX-950, a protease inhibitor) (Fig. 4E; *P* < 0.05, *vs* telaprevir alone) and IFN-α (Fig. 4F; *P* < 0.05, *vs* IFN-α alone), respectively, in HCV-infected Huh7.5 cells, indicating that combination of CD36 inhibitors with known drugs might increase the anti-HCV efficacy.

Antiviral activity of the CD36 inhibitors was also tested for their activity against hepatitis B virus, influenza virus, herpes simplex virus and Coxsackie virus in HepG2.2.15, MDCK and Vero cell cultures, respectively. The SSO showed no inhibitory activity on these viruses. Similarly, W-9 was without effect (Table 1). This result suggests that CD36 is a co-factor specific for HCV entry.

### SSO directly bound CD36 and prevented HCV from attachment on host cells

**Figure 5:**
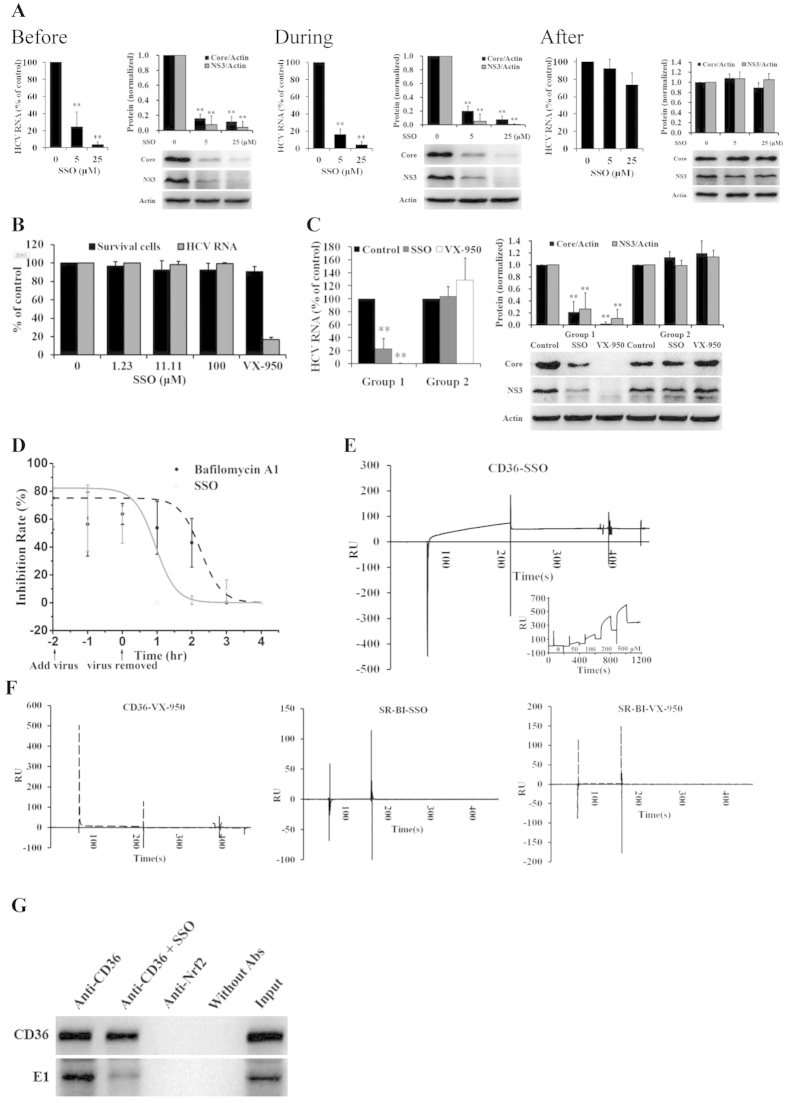
SSO directly bound CD36 and prevented HCV from attachment on host cells. (**A**) SSO only inhibited HCV replication at early stage of HCV life cycle (*n* = 3 for RNA, *n* = 4 for protein). Huh7.5 cells were treated with SSO before (at −2 hr), during (at 0 hr) or after (at 2 hr) HCV infection (150 IU/cell). After 2 hrs incubation, the supernatants were replaced with fresh culture media. Intracellular HCV RNA and proteins were detected in 72 hrs. **P < 0.01, *vs* solvent control. (**B**) SSO did not inhibit the replication of HCV sub-genome replicon in GS4.3 cells (*n* = 3). GS4.3 cells were treated with SSO or solvent control. After 72 hrs incubation, intracellular RNAs were detected, and cytotoxicity was measured with a MTT assay. VX-950 (1.0 μM) served as a positive control in the test. (**C**) SSO did not show direct inactivation on HCV (left, RNA; right, proteins) (*n* = 3 for RNA, *n* = 5 for protein). The experiment protocol is in the Method section. Intracellular HCV RNA and proteins were measured. **P < 0.01, *vs* solvent control in the same group. (**D**) Time-of-addition experiment showed that SSO prevents HCV from replication at early stage (*n* = 3). The experiment protocol is in the Method section. Intracellular HCV RNA was detected with qRT-PCR. Fitted lines represent sigmoidal time-dependent curves. (**E**) The direct interaction between CD36 and compound SSO was analyzed with BIAcore, and the interaction was in a dose-dependent manner (insert). (**F**) There was no direct interaction between CD36 and VX-950 (left), or SSO and SR-BI (middle), or VX-950 and SR-BI (right), in the BIAcore analysis. The protein bands presented showed the results of a representative experiment. Presented is mean ± standard deviation, and *Student*’s *t*-test was used in (**A–D**). (**G**) The interaction between CD36 and HCV E1 protein was disrupted by SSO. Co-IP assay was performed with the lysates of HCV-infected Huh7.5 cells and 20 μM SSO was added additionally. Anti-Nrf2 antibody was used as an irrelevant negative control, and Without Abs as blank control.

We next investigated the mechanism of SSO in its action against HCV entry. First, the compound SSO was added into cell cultures at the time of before, during and after HCV infection, respectively. Two hrs after viral infection the supernatants were removed and fresh media were added. HCV RNA and proteins were detected in 72 hrs. While SSO treatment before and during HCV infection could strongly inhibit HCV replication in Huh7.5 cells (Fig. 5A; *P* < 0.01), SSO treatment after infection showed no inhibitory effect on HCV (Fig. 5A; *P* > 0.05). It suggested that targeting CD36 by SSO might interrupt HCV replication at the attachment and entry stage.

To further validate the molecular mechanisms of SSO, GS4.3 cells, a human hepatoma Huh-7 cell line carrying an HCV subgenomic replicon I 377-3′del.S^31^, was used. Indeed, SSO did not inhibit the replication of the sub-gene HCV replicon in GS4.3 cells (Fig. 5B), as the replicon system did not have E1 protein, and viral attachment/entry steps were absent. The reference drug VX-950 showed strong inhibitory effect on replication of the HCV replicon in GS4.3 cells.

In addition, we investigated whether SSO inactivate HCV particle directly. HCV virus stock was pre-treated with SSO (10 μM) or VX-950 (0.5 μM) for 1 hr at room temperature, followed by purification through ultracentrifugation. The pellet was resuspended in fresh culture media to infect Huh7.5 cells (Group 2). SSO- or VX-950-treated HCV with no ultracentrifugation served as controls (Group 1). Intracellular HCV RNA and proteins were detected 72 hrs after infection. While compounds SSO and VX-950 showed HCV inhibitory activity in the Group 1 (Fig. 5C, Group 1, *P* < 0.01), SSO-treated viruses displayed infectivity equal to that of untreated control and negative reference VX-950 in Group 2 (Fig. 5C, Group 2, *P* > 0.05). The results indicated a good separation of SSO (and VX-950) from HCV particles in supernatants using ultracentrifugation, and null of direct interaction between SSO and HCV particles.

Then, we determined the kinetics of inhibition on HCV by SSO at the early step of viral life cycle, and a time-of-addition experiment was conducted. HCV remained to be sensitive to bafilomycin A1 (Fig. 5D), a fusion inhibitor that prevents endosome acidification, for 3 hrs after shifted to 37 °C, consistent with previous reports^32^. In contrast, SSO almost completely lost its activity when added after the temperature shift (Fig. 5D), indicating that it might act upon a very early step in viral infection.

We then analyzed whether there is a direct interaction between SSO and CD36. Purified CD36-HA protein was fixed onto chips and reacted with SSO in a mobile phase using a BIAcore T100 system. As shown in Fig. 5E, a direct binding between CD36 and SSO was detected, and the interaction was in a dose-dependent manner (Fig. 5E, insert), suggesting that the anti-attachment activity of SSO on HCV entry might be mediated via a direct binding of the compound onto CD36. And, the molecule interaction was in a covalent fashion (Fig. 5E, insert), which were consistent with the previous report^28^. As negative control, VX-950 did not bind onto CD36 (Fig. 5F, left). Also, SSO (or VX-950) showed no binding on SR-BI (Fig. 5F, middle and right), indicating a selectivity of SSO on CD36.

For detecting whether SSO will impact the interaction between CD36 and HCV E1, Co-IP assay was carried out. Result showed that SSO could directly disrupt the interaction between CD36 and HCV E1 (Fig. 5G), suggesting that the binding of SSO to CD36 will disrupt the direct interaction between CD36 and HCV E1.

### CD36 inhibitors were safe *in vivo*

**Figure 6:**
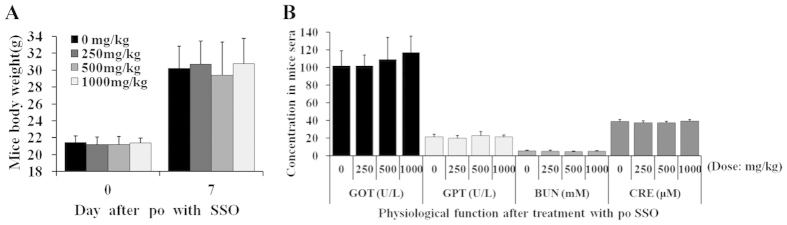
Safety of SSO in mice. Oral administration of SSO (single dosing) in mice (*n* = 10; ♂ × 5 and ♀ × 5) caused no change in survival and body weight (**A**); liver and kidney function was without change as well (**B**). GOT, glutamate-oxaloacetate transaminase (U/L); GPT, glutamate-pyruvate transaminase (U/L); BUN, blood urea nitrogen (mM); CRE, creatine (μM).

Normal KM mice were orally treated with a single dose of SSO in a range between 250 to 1000 mg/kg, and the mice were followed up for 7 days. As shown in Fig. 6, none of the mice died in the groups treated with SSO, and body weight change was not observed (Fig. 6A). Blood samples were taken at the end of the experiment and liver as well as kidney functions were examined. Even at a dose up to 1000 mg/kg, abnormality was not detected in blood GOT, GPT, BUN and CRE (Fig. 6B), suggesting that CD36 inhibitor SSO was relatively safe *in vivo*. Considering that SSO strongly inhibited HCV replication and was with high SI in hepatocytes, CD36 might a potential target to develop new mechanism anti-HCV agents.

## Discussion

CD36, also known as fatty acid translocase (FAT), is an integral membrane glycoprotein and belongs to the class B scavenger receptor family which includes receptors for selective cholesteryl ester uptake, scavenger receptor class B type I (SR-BI) and lysosomal integral membrane protein II (LIMP-II)^33^. CD36 combines with a variety of ligands and plays an important role in various physiological and pathological processes, such as the formation of macrophage foam cells, atherosclerosis, angiogenesis, diabetes mellitus, thrombosis, malaria, Alzheimer’s disease, obesity, and tumorigenesis as well, and thus may be a potential target to drug discovery^20^,^34^. Soluble CD36 (sCD36), a cell-free form of CD36^35^, is significantly associated with indices of insulin resistance, carotid atherosclerosis and fatty liver^36^. Clinical study showed an increased expression of CD36 on liver cells and elevated blood level of sCD36 in subjects infected with HCV^23^,^24^,^25^, however, the biological significance remains unclear. We showed in this study that HCV infection increased expression of CD36 in hepatocytes and elevated secretion of sCD36 in cell culture supernatants, confirming the clinical observation mentioned above. However, Himoto T *et al.* showed that the loads of HCV RNA did not affect the serum sCD36 levels in patients with HCV-related chronic liver disease, and they conjectured that sCD36 levels are regulated by host factors rather than the viral factors themselves^24^. It is reported that HCV core protein increases hepatic lipid accumulation via elevating transcriptional activity of peroxisome proliferators-activated receptor gamma (PPAR-gamma)^37^, which is important in regulation of a number of genes involved in hepatic fatty acid synthesis, and its transcriptional activation up-regulates expression of CD36^38^,^39^,^40^. So the level of CD36 (or sCD36) was up-regulated by HCV infection might be through the increase of PPAR-gamma transcriptional activity caused by HCV Core. The detailed mechanism needs to be further clarified.

The membrane CD36 contributed to the HCV replication through facilitating the viral attachment on host cell membrane. Molecular mechanism study showed that HCV E1 protein on the surface of viral particles could directly bind membrane CD36, helping HCV to complete its entry process. The sCD36 increased HCV entry as well, but the mechanism is not understood. There could be at least two explanations: first, binding of sCD36 with its ligand (HCV E1) might induce formation of CD36 dimers or multimers with those on membrane^41^; or/and second, sCD36 in a complex with HCV E1 might increase adherence on cell surface through interacting with membrane proteins^42^. Our results showed that HCV infection causes an increased expression and secretion of CD36, and in turn, the elevation of CD36 (or sCD36) would facilitate HCV replication. Normally, virus infection could prevent superinfection. HCV infection of Huh 7 cells down-regulated the expression of CLDN1 and occludin, and thus prevented superinfection^43^. However, HCV superinfection exclusion was neither due to a reduction of cell surface expression of CD81 and scavenger receptor BI, two molecules implicated in HCV entry, nor due to a functional block at the level of virus entry^44^. The mechanism and consequence is still to be clarified, especially *in vivo*. sCD36 is a cell-free form of CD36 showing positive correlation with membrane CD36 expression and HCV replication, thus it might be a biomarker in HCV infected individuals.

CD36 has a protein sequence with 30% homology to that of SR-BI^27^, a known host factor for HCV entry^6^. Thus, three CD36-specific mAbs were used in these study, and we found that two out of three were active against HCV entry; combination of CD36 mAb with SR-BI antibody showed additional inhibition on HCV replication. The results suggested that CD36 contributes to HCV entry via a mode of action independent of that by SR-BI. Indeed, CD36 and SR-BI binds different proteins of HCV. A previous report showed that SR-BI selectively bound HCV E2, but CD36 did not^6^; and we show here that CD36, but not SR-BI, bound HCV E1. As HCV entry requires presence of both E1 and E2 proteins on viral surface^45^,^46^, it explains the additional anti-HCV effect when the CD36 mAb was used in combination with SR-BI antibody.

CD36 is expressed on several types of mammalian cells and has multiple effects in biology^20^, especially in lipid metabolism. Thus, one of the concerns was that whether inhibition of CD36 will hurt physiological functions *in vivo*. Independent studies of several groups have shown that deficiency or null of CD36 was not lethal, and caused only metabolic disorders in rat or in human^47^,^48^, suggesting a promise in safety. Thus, it is suggested that CD36 may be a potential drug target for lipid metabolism^28^,^29^,^30^,^49^.

For many years, interferon (IFN-alpha) in combination with ribavirin has represented a standard therapy of HCV infection, although the efficacy is not satisfactory^50^. Recently, several DAAs were approved for clinic use^51^, including NS3/4A serine protease inhibitors telaprevir, boceprevir and simeprevir, as well as NS5B polymerase inhibitor sofosbuvir. These drugs have improved the overall therapeutic efficacy on hepatitis C. However, as the agents target specific HCV enzymes, emergence of drug-resistant viral strains could be a hurdle in near future^52^,^53^. To address the concern, research efforts have been mainly focused on combination therapy as well as new mechanism drugs inhibiting viruses through host factors^54^,^55^. Drugs that inhibit virus via regulating host environment might have less chance of inducing drug-resistant mutation^56^.

In conclusion, our results showed that membrane protein CD36 expressed in liver cells appears to be a co-receptor for binding with HCV E1 protein in the attachment stage. Blocking CD36 with its inhibitors significantly interrupts HCV replication. We consider CD36 a potential drug target against HCV.

## Materials and Methods

### Cells and HCV

Huh7.5 cells and the plasmid pFL-J6/JFH/JC1 containing the full-length chimeric HCV complementary DNA (cDNA) were kindly provided by Vertex Pharmaceuticals, Inc. HCV virus stock was prepared as previously described^57^. 293T/17 cells (from ATCC) and GS4.3 replicon cells were cultured as described previously^58^,^59^.

### Agents

Sulfo-N-succinimidyl oleate (SSO, sc-208408), bafilomycin A1 (sc-201550) and siRNAs for CD36 (sc-29995), SR-BI (sc-44752 and sc-44753) and negative control (siRNA-A, sc-37007) were from Santa Cruz Biotechnology, Inc. The compounds W-9 and AP5055 were synthesized in the Medicinal Chemistry Laboratory of Institute of Medicinal Biotechnology, Chinese Academy of Medical Sciences, in a purity greater than 98.5%. The structures of the compounds were confirmed with proton nuclear magnetic resonance spectroscopy and mass spectrometry spectra. Telaprevir/VX-950 (HY-10235) was from MedChemExpress, Inc. The antibody to SR-BI (NB400-104) was from Novus Biological, Inc. The mAbs to human CD36 (ab17044, ab23680, ab76521 and ab133625), HCV core (ab2740), HCV NS3 (ab13830), control IgG_1_ (ab18447) and Nrf2 antibody (ab31163) were from Abcam, Co. Ltd. The mAb to beta-Actin (TA-09) was from Beijing ZSJQ-BIO, Co. Ltd. The antibodies to HCV E1 (GTX103352) and E2 (GTX103353) were from Gene Tex, Inc. The mAb to HA tag (6E2) (2367S) and the secondary antibodies (7074S and 7076S) were from Cell Signaling Technology, Inc.

### Plasmids

The plasmids pcDNA3.1(+)-CD36 expressing CD36, pcDNA3.1(+)-CD36-HA expressing CD36 with HA tag at the C-terminal, and pZeoSV2(+)-SR-BI-HA expressing SR-BI with HA tag at C-terminal were cloned from Huh7.5 cell with primers shown in Table 2. The sequences were confirmed by sequencing and assay of restriction endonucleases respectively.

### CD36 expression in HCV-infected cells

Huh7.5 cells were inoculated with HCV (45 IU/cell). At 0, 2 and 4, or over 60 days after infection, the culture supernatants were harvested to extract proteins with Protein Recovery Kit (Applygen Technologies, Inc.). Intracellular proteins and total membrane proteins were extracted with the RIPA Lysis Buffer containing 1 mM protease inhibitor cocktail (Roche Applied Science) and Thermo MEM-PER Eukaryotic Membrane Protein Extraction Reagent Kit (Thermo Scientific) respectively, according to the manufacturers’ instructions. Proteins analysis was done with western blot (WB).

### Over expression and silence

Huh7.5 cells were transfected with 0.2 μg CD36-HA plasmid or plasmid control pcDNA3.1(+) using HD transfection reagent (Promega), or transfected with 150 pmol siRNA for CD36 or SR-BI or negative control siRNA-A using RNAiMAX transfection reagent (Invitrogen). After 48 hrs, the culture supernatants were harvested to extract proteins or to treat naïve Huh7.5 cells. The transfected cells were washed and then infected with HCV (150 IU/cell) for 2 hrs, followed by washing and culturing with fresh medium. After 24 hrs, the cells were subcultured, or intracellular proteins were extracted to detect CD36 expression with WB. Naïve Huh7.5 cells were cultured with 2 ml of above culture supernatants and simultaneously infected with HCV (150 IU/cell) for 2 hrs, followed by washing and culturing with fresh medium. 72 hrs later, the intracellular HCV RNA was extracted with RNeasy Mini Kit (Qiagen) and total intracellular proteins were extracted with PER (CytoBuster Protein Extraction Reagent (Novagen) with 1 mM protease inhibitor cocktail). HCV RNA was quantified with real-time quantitative RT-PCR (qRT-PCR), and proteins were analyzed with WB. siRNAs cytotoxicity in Huh7.5 cells was evaluated accordingly with the tetrazolium (MTT, Amresco) assay^57^.

### Anti-HCV activities of compounds *in vitro*

Huh7.5 cells infected with HCV (45 IU/cell), or GS4.3 replicon cells were treated with different concentration of compounds. After 72 hrs, intracellular RNA was extracted with RNeasy Mini Kit; total intracellular proteins were extracted with PER. HCV RNA and proteins were analyzed with qRT-PCR and WB, respectively. Compound cytotoxicity in Huh7.5 cells or GS4.3 replicon cells were evaluated at 72 hrs with the tetrazolium (MTT, Amresco) assay^57^.

### Specific mAb test

Huh7.5 cells were treated with mAb at 5 μg/mL for 1 hr, and then infected with HCV (150 IU/cell). After 2 hrs infection, the cells were washed with PBS and cultured with fresh medium. 72 hrs later, intracellular HCV RNA was extracted and quantified with qRT-PCR.

### Immunoprecipitation assay

HCV infected Huh7.5 cells were collected and washed with DPBS, the agglomerates were lysed in 1mL PER and then centrifuged at 12000 × g for 20 min at 4 °C. The supernatants were then incubated with 4 μg anti-CD36 mAb (ab133625) for 16 hrs at 4 °C. After adding 75 μL of Protein G Agarose (Roche Applied Science), the mixture was incubated for other 3 hrs; irrelevant antibody (ab31163) was used as negative control. The proteins in the immuno-precipitate were analyzed with WB. For detecting the role of SSO in the binding between CD36 and HCV E1, 20 μM SSO was added additionally in the Co-IP assay.

### The influence of CD36 on the anti-HCV activity of SSO

Huh7.5 cells were transfected with 0.2 μg CD36 plasmid or plasmid control pcDNA3.1(+), or transfected with 150 pmol siRNA for CD36 or negative control siRNA-A. After 48 hrs, the transfected cells were washed and then infected with HCV (150 IU/cell) and simultaneously treated with 5 μM SSO or solvent control for 2 hrs, followed by washing and culturing with fresh media. Intracellular HCV RNA was analyzed with qRT-PCR in 96 hrs.

### Inhibition of HCV entry

Huh7.5 cells were infected with HCV (150 IU/cell) and treated with SSO for 2 hrs prior to infection (Before), during the 2 hrs infection (During), or immediately after infection for 2 hrs (After). Then, the cells were washed and cultured with fresh medium. Intracellular HCV RNA and proteins were detected in 72 hrs.

### qRT-PCR

The total RNA extracted from Huh7.5 cells was analyzed using the AgPath-ID One-Step RT-PCR Kit (Applied Biosystems, Foster, CA), Fluorescent signals were detected with 7500 fast real-time PCR system (Applied Biosystems) according with manufacturer’s procedure. The levels of HCV RNA were analyzed with the 2^−△△CT^ method, and all quantifications were normalized to the level of the internal control gene, glyceraldehyde 3-phosphate dehydrogenase (GAPDH)^60^, The sequences of primer pairs and probes for HCV RNA and GAPDH were shown in Table 2.

### Western blot

The western blot was performed as previously described^57^. After SDS-PAGE and trans-Membrane, the target proteins were accordingly probed with anti-HCV Core antibody (1:2500), or anti-HCV NS3 antibody (1:2500), or anti-CD36 antibody (ab133625, 1:2500) or anti-SR-BI antibody (1:2500), or anti-HA tag antibody (1:1000), respectively. As a control, anti-Actin antibody (1:4000) was used. The proteins were detected using Immobilon Western Chemiluminescent HRP Substrate (Millipore, Inc.) with Alpha Innotech Focus and Image Acquisition (Alpha Innotech, Inc.). The protein signal intensity was scanned with Gelpro32 software, the ratio of interested protein to internal control protein Actin was calculated, and normalized as 1.00 for the control group.

### BIAcore

The 293T/17 cells were transfected with CD36-HA or SR-BI-HA plasmid using HD transfection reagent. At 48 hrs post transfection, cells were harvested and protein CD36-HA or SR-BI-HA was purified with Pierce Anti-HA Agarose (Thermo Scientific) and then desalinated with Zeba Spin Desaling Columns (Thermo Scientific). The concentration of purified proteins was quantified with Pierce BCA Protein Assay Kit (Thermo Scientific). Then, surface plasmon resonance experiments were performed on a BIAcore biosensor system (BIAcore T100). In brief, the carboxymethylated surface of series S sensor chip CM5 (GE Healthcare Bio-Sciences) was first activated with a mixture of N-hydroxysuccinimide (NHS) and 1-ethyl-3-(3- dimethylaminopropyl) carbodiimide hydro-chloride (EDC) (BIAcore AB). Subsequently, 200 μL of purified CD36-HA or SR-BI-HA at a concentration of 80 μg/mL was injected into the flow cell (FC2) for immobilization on the sensor surface. To analyze the binding of SSO with CD36 or SR-BI protein, 160 μL of SSO [50, 100, 200, 500 μM in HBS-EP + buffer (GE Healthcare Bio-Sciences)] were injected and followed by flowing the buffer. Compound VX-950 was used as negative control. Data were evaluated using the software BIAcore T100 Evaluation (BIAcore AB).

### Interaction between HCV and compounds

HCV viral stock was treated with SSO (10 μM) or VX-950 (0.5 μM) or solvent control for 1 hr at room temperature. Group 1 was not centrifuged and used as control; Group 2 was ultracentrifuged at 30000 × g for 4 hrs at 4 °C, and the pellet was resuspended with complete culture medium. Huh7.5 cells were infected with above treated-HCV (45 IU/cell), and intracellular HCV RNA and proteins were detected in 72 hrs.

### Time-of-addition assay

Huh7.5 cells were infected with HCV (300 IU/cell) and incubated at 4 °C for 2 hrs, the cells were then washed with cold PBS and shifted to 37 °C (set as 0 hr time point) to initiate synchronous infection. At −2, −1, 0, 1, 2, 3, 4 hr, 10 μM SSO, or 10 nM bafilomycin A1, or solvent control was added, and the cells were incubated for 2 hrs prior to removal (the exception is t = −2 hr and −1 hr where compound were added back after removal of the virus and incubated for additional 2 hrs prior to removal). Intracellular HCV RNA was detected after 72 hrs incubation. Inhibitory ratio was calculated as % relative to infectious control (0%). Fitted lines represent sigmoidal time-dependent curves^32^.

### Interaction between HCV and sCD36

Culture supernatants were collected from HCV infected Huh7.5 cells and centrifuged at 1000 × g for 10 minutes at 4 °C, and then ultracentrifuged at 30000 × g for 1 hr at 4 °C. Equal amounts of culture media from naïve Huh7.5 cells were used as negative control. The proteins in precipitates were detected with WB.

### 293T/17 cell line model

293T/17 cells were transfected with 1 μg CD36 plasmid or plasmid control pcDNA3.1(+) using HD transfection reagent, or transfected with 150 pmol siRNA for CD36 or control siRNA-A using RNAiMAX transfection reagent. 48 hrs later, the cells were washed with PBS and then infected with HCV (300 IU/cell) for 3 hrs at 37 °C. After washing 3 times with PBS, the cells were lysed with PER and proteins were detected with WB.

### Safety of SSO *in vivo*

KM mice at a weight of 21.0 ± 1.0 g were from Laboratory Animal Center, Academy of Military Medical Sciences, Beijing, China. The mice were randomly divided into four groups with 10 mice in each (♂ × 5, ♀ × 5). The mice were treated with intragastric administration of 0.4 ml of SSO suspension at a single dose of 0 (5% Tween-80 as control), 250, 500, 1000 mg/kg, respectively. Body weight as well as survival was monitored. Seven days later, blood samples were taken for liver and kidney function examination.

### Ethics Statement

All animals were housed in the animal facility of the Institute of Medicinal Biotechnology, Chinese Academy of Medical Sciences, Beijing. Animal experiments were conducted following the National Guidelines for Housing and Care of Laboratory Animals and performed in accordance with protocol after approved by the Institutional Animal Care and Use Committee (Protocol Number: SYXK(Jing)2012-0021).

### Statistical analysis

Data shown in the histogram were mean ± standard deviation of over 3 independent experiments. Data were analyzed using ANOVA analysis and *Student*’s *t*-test. The level of significance was set at *P* < 0.05.

## Additional Information

**How to cite this article**: Cheng, J.-J. *et al.* CD36 is a co-receptor for hepatitis C virus E1 protein attachment. *Sci. Rep.* **6**, 21808; doi: 10.1038/srep21808 (2016).

## Figures and Tables

**Table 1:** The activity of SSO against other viruses.

	HBV	HSV-1	CoxV-B1	Influenza virus	
				A/Tianjin-Jinnan/15/2009	BV/Shenzhen/155/2005
**CC**_**50**_(μ**M)**	>100	>200	111.47	>200	>200
**EC**_**50**_(μ**M)**	>100	>200	>66.67	79.0	200
**SI**	—	—	<1.73	>2.53	>1.0

Note: The activity of SSO against hepatitis B virus (HBV) were tested in HepG2.2.15, herpes simplex virus type 1 (HSV-1) and Coxsackie B virus (CoxV-B1) were tested in Vero, and influenza virus were tested in MDCK cells. EC_50_, half maximal effective concentration. CC_50_, half maximal cytotoxicity concentration.

**Table 2:** The primers and probes used in PCR or qRT-PCR.

Gene	Primer pairs	Probe
CD36	5′-CCAAGCTTATGGGCTGTGACCGGAACT-3′	
	5′-CCGGAATTCTTATTTTATTGTTTTCGATCTGCATGC-3′	
CD36-HA	5′-CCAAGCTTATGGGCTGTGACCGGAACT-3′	
	5′-CCGGAATTCTTAAGCGTAATCTGGAACATCGTATGGGTATTTTATTGTTTTCGATCTGCATGC-3′	
SR-BI-HA	5′-CTAGCTAGCATGGGCTGCTCCGCCAAAG-3′	
	5′-CCAAGCTTCTAAGCGTAATCTGGAACATCGTATGGGTACAGTTTTGCTTCCTGCAGCACAG-3′	
HCV	5′-CGGGAGAGCCATAGTGGTCTGCG-3′	FAM-5′-AGGCCTTGTGGTACTGCCT-3′-TAMRA
	5′-CTCGCAAGCACCCTATCAGGCAGTA-3′	
GAPDH	5′-CGGAGTCAACGGATTTGGTCGTAT-3′	FAM-5′-CCGTCAAGGCTGAGAACGG-3′-TAMRA
	5′-AGCCTTCTCCATGGTGGTGAAGAC-3′	

Note: underline showed the sequence of restriction endonucleases sites.
